# The complete chloroplast genome sequence of *Paris polyphylla* var. *alba* H.Li & R.J.Mitchell and its phylogenetic analysis

**DOI:** 10.1080/23802359.2021.1959450

**Published:** 2021-07-27

**Authors:** Yuan Jiang, Qingshu Yang, Jing Wang, Jun Qian, Baozhong Duan

**Affiliations:** a College of Pharmaceutical Science, Dali University, Dali, China; b Key Laboratory of Yunnan Provincial Higher Education Institutions for Development of Yunnan Daodi Medicinal Materials Resources, Yunnan, China

**Keywords:** *Paris polyphylla* var. *alba* H.Li & R.J.Mitchell, complete chloroplast genome, phylogeny, Melanthiaceae

## Abstract

*Paris polyphylla* var. *alba* is a medicinal plant commonly used in the southwest of China. This study characterized the complete chloroplast (cp) genome sequence of *P. polyphylla* var. *alba* to investigate its phylogenetic relationship in Melanthiaceae. The cp genome of *P. polyphylla* var. *alba* is 165,079 base pairs (bp) in length with 36.96% G + C content. The cp genome is divided into (a) large single copy (LSC) (84,393 bp), (b) small single copy (SSC) (16,066 bp), and (c) two inverted regions (32,310 bp). The cp genome contains 135 genes, including 89 protein-coding genes, 38 tRNA genes, and 8 rRNA genes. Phylogenetic analysis indicated that *P. polyphylla* var. *alba* is closest to *P. polyphylla* var. *emeiensis*, and *Paris* had a close relationship with *Trillium* in Melanthiaceae.

*Paris polyphylla* var. *alba* H.Li & R.J.Mitchell is a flowering herb belonging to the *Paris* genus in the Melanthiaceae family, first described by Li Heng in 1986 (Li 1986; Wu et al. 2018). The dried rhizome has been widely used in traditional Chinese medicine for the treatment of hemostatic, sore throat, snake bite, and convulsion, etc. (Duan et al. 2018, Fu et al. 2012, Liu et al. 2017). To date, the studies of this species have mainly focused on its pharmacological activity, chemical composition, and quantitative analysis (Yin et al. 2007, Zhe et al. 2017). However, no data are available regarding the genomic studies on *P. polyphylla* var. *alba* and its relationship with other species belonging to *Paris*. Herein, the cp genome of *Paris polyphylla* var. *alba was* assembled and characterized for the first time, which will provide helpful information for further study of the genus.

Fresh leaves of *P. polyphylla* var. *alba* collected from Germplasm Resource Garden of Longmen Township (Yunan, China, 25°32′38″N, 99°32′8″E). A specimen was deposited at the herbarium of Dali University (https://www.dali.edu.cn/, Baozhong Duan and bzduan@126.com) under the voucher number 20200820A11. A total genomic DNA of sample was extracted with plant genomic DNA kit (Tiangen Biotech, China) and sequenced by using the Hiseq 2500 platform (Illumina, San Diego, CA). Approximately 4.15 Gb of raw data (26,636,980 reads) was assembled by NOVOPlasty (Nicolas et al. 2017), and annotated by CPGAVAS2 (Shi et al. 2019). Annotated cp genome sequence was submitted to GenBank under accession number no. MW727455.

The cp genome sequence of *P. polyphylla* var. *alba* is 165,079 bp in length, with a large single-copy region (LSC) of 84,393 bp, a small single-copy region (SSC) of 16,066 bp, and a pair of inverted repeats (IR) regions of 32,310 bp. GC content of the whole genome, LSC, SSC, and IRs regions are 36.96%, 35.65%, 31.57%, and 40.00%, respectively. A total of 135 genes were annotated, including 89 protein-coding genes, 38 tRNA genes, and 8 rRNA genes. The gene content and organization of the inverted repeat are similar to the other members of *Paris* (Fan et al. 2020).

To reveal the phylogenetic position of *P. polyphylla* var. *alba* with other members of Melanthiaceae, a phylogenetic analysis was performed based on 23 complete cp genomes. In addition, *Lavandula angustifolia* (NC_029370) and *Mentha Canadensis* (NC_044082) were downloaded and included as an outgroup. The MAFFT v7.307 was used to extract the coding sequences, and a total of 81 coding sequences (*acc*D, *atp*A, *atp*B, *atp*E, *atp*F, *atp*H, *atp*I, *ccs*A, *cem*A, *clp*P, *inf*A, *lhb*A, *mat*K, *ndh*A, *ndh*B, *ndh*C, *ndh*D, *ndh*E, *ndh*F, *ndh*G, *ndh*H, *ndh*I, *ndh*J, *ndh*K, *pet*A, *pet*B, *pet*D, *pet*G, *pet*L, *pet*N, *psa*A, *psa*B, *psa*C, *psa*I, *psa*J, *psb*A, *psb*B, *psb*C, *psb*D, *psb*E, *psb*F, *psb*H, *psb*I, *psb*J, *psb*K, *psb*L, *psb*M, *psb*N, *psb*T, *psb*Z, *rpc*L, *rpl*2, *rpl*14, *rpl*16, *rpl*20, *rpl*22, *rpl*23, *rpl*32, *rpl*33, *rpl*36, *rpo*A, *rpo*B, *rpo*C1, *rpo*C2, *rps*2, *rps*3, *rps*4, *rps*7, *rps*8, *rps*11, *rps*12, *rps*14, *rps*15, *rps*16, *rps*18, *rps*19, *ycf*1, *ycf*2, *ycf*3, *ycf*4, *ycf*15) were presented in all of the 24 species. Then the MAFFT v7.307 was used to concatenate the coding sequences and align the concatenation sequences. Afterward, RAxML (version 8.2.12) (Stamatakis 2014) was used to construct the maximum likelihood (ML) tree; bootstrap probability values were calculated from 1000 replicates. The ML tree showed that *P. polyphylla* var. *alba*, *P. polyphylla* var. *emeiensis*, *P. liiana*, and *P. polyphylla* were clustered together (Figure 1). The work reported the first complete cp genome of *P. polyphylla* var. *alba,* which will provide further insight into the evolution of Melanthiaceae.

**Figure 1.**
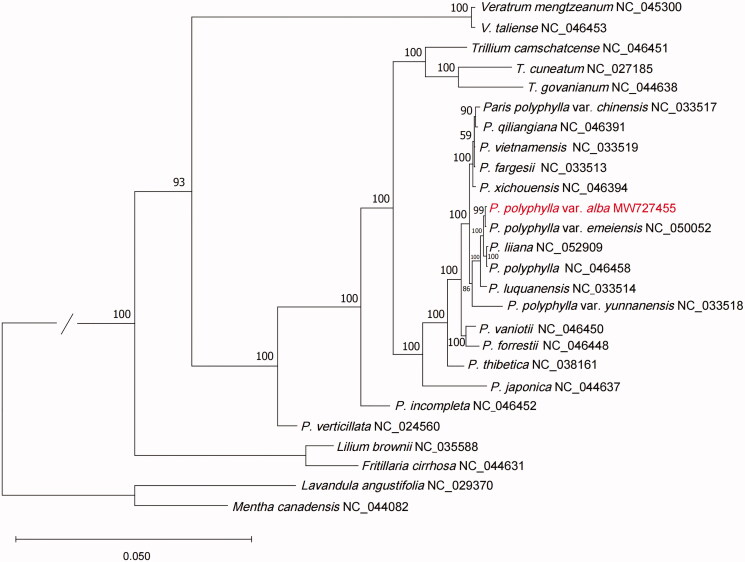
Phylogenetic analysis of 24 species and two taxa as outgroups based on cp genome sequences by RAxML, bootstrap support value near the branch.

## Data Availability

The data that support the findings of this study are openly available in GenBank at https: https://www.ncbi.nlm.nih.gov/nuccore/MW727455, Associated BioProject, https://www.ncbi.nlm.nih.gov/bioproject/PRJNA728587, BioSample accession number at https://www.ncbi.nlm.nih.gov/biosample/SAMN19092376 and Sequence Read Archive at https://www.ncbi.nlm.nih.gov/sra/SRR14506499.
